# Transcriptional profiling of *Actinobacillus pleuropneumoniae *under iron-restricted conditions

**DOI:** 10.1186/1471-2164-8-72

**Published:** 2007-03-13

**Authors:** Vincent Deslandes, John HE Nash, Josée Harel, James W Coulton, Mario Jacques

**Affiliations:** 1 Groupe de Recherche sur les Maladies Infectieuses du Porc, Département de Pathologie et Microbiologie, Faculté de Médecine Vétérinaire, Université de Montréal, St-Hyacinthe, Québec, J2S 7C6, Canada; 2 Institute for Biological Sciences, National Research Council of Canada, Ottawa, Ontario, K1A 0R6, Canada; 3 Department of Microbiology and Immunology, McGill University, Montréal, Québec, H3A 2B4, Canada; 4 Canadian Research Network on Bacterial Pathogens of Swine (SIDNet), St-Hyacinthe, Québec, Canada

## Abstract

**Background:**

To better understand effects of iron restriction on *Actinobacillus pleuropneumoniae *and to identify new potential vaccine targets, we conducted transcript profiling studies using a DNA microarray containing all 2025 ORFs of the genome of *A. pleuropneumoniae *serotype 5b strain L20. This is the first study involving the use of microarray technology to monitor the transcriptome of *A. pleuropneumoniae *grown under iron restriction.

**Results:**

Upon comparing growth of this pathogen in iron-sufficient *versus *iron-depleted medium, 210 genes were identified as being differentially expressed. Some genes (92) were identified as being up-regulated; many have confirmed or putative roles in iron acquisition, such as the genes coding for two TonB energy-transducing proteins and the hemoglobin receptor HgbA. Transcript profiling also led to identification of some new iron acquisition systems of *A. pleuropneumoniae*. Genes coding for a possible Yfe system (*yfeABCD*), implicated in the acquisition of chelated iron, were detected, as well as genes coding for a putative enterobactin-type siderophore receptor system. ORFs for homologs of the HmbR system of *Neisseria meningitidis *involved in iron acquisition from hemoglobin were significantly up-regulated. Down-regulated genes included many that encode proteins containing Fe-S clusters or that use heme as a cofactor. Supplementation of the culture medium with exogenous iron re-established the expression level of these genes.

**Conclusion:**

We have used transcriptional profiling to generate a list of genes showing differential expression during iron restriction. This strategy enabled us to gain a better understanding of the metabolic changes occurring in response to this stress. Many new potential iron acquisition systems were identified, and further studies will have to be conducted to establish their role during iron restriction.

## Background

*Actinobacillus pleuropneumoniae*, etiological agent of porcine pleuropneumonia, causes great commercial losses to the swine industry worldwide [1]. Transmission of this highly contagious disease that affects pigs of all ages occurs mostly by aerosol and close contact with infected animals [2]. During 24 to 48 hours of the acute phase of the disease, formation of extensive and fibrinohemorrhagic lung lesions is often fatal. Animals that survive the disease may become asymptomatic carriers of the bacteria, developing localized and necrotizing lesions associated with pleuritis [3]. Based on differences of capsular polysaccharides, fifteen serotypes have been identified: serotypes 1 to 12 and 15 belong to biotype 1, which is NAD-dependent; serotypes 13 and 14 are classified in biotype 2, which is NAD-independent [4]. In North America, serotypes 1, 5 and 7 are prevalent, while serotypes 2 and 9 are most commonly found in Europe.

Despite many years of research, the total complement of bacterial components that are involved in infection by *A. pleuropneumoniae *has yet to be identified. Several virulence factors have been proposed: capsular polysaccharides, lipopolysaccharides (LPS), Apx toxins and various iron acquisitions systems [2]. However, the overall contribution of each component to the infection process remains unclear. Although less virulent, an acapsular mutant was still serum-resistant, showed higher adhesion to piglet tracheal frozen sections and could still be re-isolated from lungs of infected animals [5]. LPS apparently plays a role in adhesion *in vivo*, as these molecules show *in vitro *adhesion to many biological components [4]. The Apx toxins contribute to development of lesions typically associated with the disease [6] and mutants missing Apx toxins are avirulent in pigs and mice [7]. However, different *A. pleuropneumoniae *serotypes secrete different sets of Apx toxins, and the relative contribution of the four different Apx toxins (ApxI to IV) is still not clear.

Low availability of iron in the host represents a major stress for bacterial pathogens and is considered a signal that leads to significant changes in cell processes. Iron atoms are often linked with sulphur in Fe-S clusters in the catalytic core of enzymes involved in diverse functions such as respiration, ATP generation, and DNA replication and repair, which might account for this phenomenon. Iron is an essential element for almost every living organism. However in the host, molecules such as transferrin, lactoferrin, haptoglobin and hemoglobin in extra-cellular fluids bind free iron and iron-containing molecules very tightly [8]. While bacteria generally need free iron concentrations of about 10^-7 ^M, its concentration may be 10^-24 ^M in the mammalian host [9]. To counteract the effect of these iron-withholding mechanisms of the host, bacteria have evolved different iron acquisition systems, often relying on the secretion of siderophores, small (<1000 Da) molecules with high affinity for iron, or on surface receptors specific for iron-containing host proteins [10]. Studies in our laboratory have led to the identification, expression and characterization of the *A. pleuropneumoniae *hydroxamate siderophore receptor FhuA [11,12] and a hemoglobin binding receptor HgbA [13]. *A. pleuropneumoniae *also possesses a transferrin receptor complex composed of two outer membrane (OM) proteins: a 100 kDa TbpA may form a transmembrane channel enabling transport of iron across the OM; a 60 kDa lipoprotein TbpB acts as an auxillary molecule [2,14,15]. Energization of these OM transporters relies on the transduction of the proton motive force from the cytoplasmic membrane (CM) by the TonB-ExbB-ExbD complex [16] that is anchored in the CM and spans the periplasm. In *A. pleuropneumoniae*, two different TonB systems have been identified: genes *tonB1*-*exbB1*-*exbD1 *are transcriptionally linked to the *tbpA*-*tbpB *genes [17]; and a second system with genes *tonB*-*exbB2*-*exbD2 *was also identified [18]. Transport of iron across the CM is apparently accomplished by the AfuABC ABC transporter [19]. It has also been shown that *A. pleuropneumoniae *can use exogenous siderophores and may be able to secrete an iron chelator in response to iron stress [20].

The ferric uptake regulator Fur protein has been identified in many pathogenic bacteria, including *A. pleuropneumoniae *[21]. Using Fe^2+ ^as a cofactor, the Fur protein can interact with a specific sequence termed the Fur box in the promoter region of genes implicated in iron acquisition processes, thereby repressing transcription. When iron becomes scarce, the Fur protein loses its cofactor and becomes inactive. The fact that transcription of some genes seems to be under positive control of active Fur protein [22,23] was recently explained by the discovery of RyhB, a small non-coding RNA which belongs to the Fur regulon [24]. When transcribed, the RyhB RNA down-regulates the mRNA level of those genes that seemed to be positively regulated by Fur.

To better understand the mechanisms used by *A. pleuropneumoniae *that address iron restriction and to gain insights into strategies used by this pathogen under conditions mimicking the *in vivo *environment, we evaluated gene expression profiles of *A. pleuropneumoniae *grown under iron restriction. Our study identified 210 differentially expressed genes, of which 92 are up-regulated. Within the latter set, components of previously unrecognized iron acquisition systems were identified: a putative enterochelin-like siderophore receptor, a potential Yfe system for the acquisition of chelated iron, a putative hemoglobin acquisition system homologous to the *N. meningitidis *HmbR system, and a putative Fe^2+^-specific porin system.

## Results and Discussion

### Microarray analysis of mRNA levels during growth of *A. pleuropneumoniae *under iron-restricted conditions

To assess the response of *A. pleuropneumoniae *to iron restriction, the reference strain S4074 was grown in BHI broth containing 50 μg/ml EDDHA, a concentration sufficient to cause iron restriction [11]. This strain was chosen because it is the strain that has been the most studied over time, but also because major problems were encountered with RNA extraction from strain L20. Preliminary CGH studies conducted in our lab showed that 95% of the genes of the *A. pleuropneumoniae *5b L20 genome are conserved between both strains. Growth curves established the optimum growth phase for RNA extraction (data not shown). At 50 μg/ml of EDDHA, bacterial growth is almost completely inhibited within an hour of addition. By adding the iron chelator at an optical density of 0.1, iron-restricted cultures and iron-rich cultures were harvested concurrently at an optical density of 0.3. Under these growth conditions, we identified 210 differentially expressed genes, with an estimated false discovery rate (FDR) of 3.22%: 118 were down-regulated (Table 1) and 92 were up-regulated (Table 2). In order to confirm that these variations were not caused by the chelator, control experiments where iron was supplemented to the restricted medium were conducted. Exogenous iron, in the form of FeCl_3_, was added to a final concentration of 50 μg/ml to the iron-depleted medium. Growth curves indicated that this concentration of FeCl_3 _was sufficient to promote growth at a similar level as in the BHI broth. Under these conditions, the expression pattern was highly similar to that seen in BHI broth: we identified only 30 differentially expressed genes, out of 2025, with an estimated FDR of 2.5%, 26 of which were up-regulated, while only 4 were down-regulated (data not-shown). Only 12 genes significantly differentially expressed in the iron-supplemented medium were identified as such in the iron-depleted *versus *BHI broth experiment, but with reversed levels of variation. Gene *lldD *(ap2032), which was up-regulated in the iron-depleted medium, was down-regulated in the iron-supplemented medium. Conversely, 11 genes that were down-regulated in the iron-depleted medium were up-regulated in the iron-supplemented medium. This indicates that the results obtained in the iron-depleted versus BHI broth experiment can be attributed to iron restriction, and not to another effect of the chelator.

### Validation of microarray results by qRT-PCR

**Figure 1:**
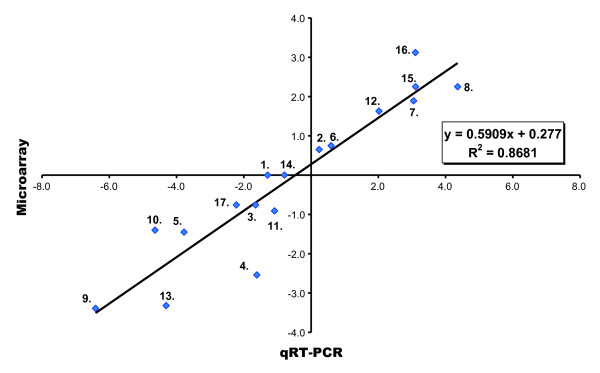
**Validation of microarray results by qRT-PCR**. Seven up-regulated genes, eight down-regulated genes and two genes that did not show significant variation in the microarray experiments are presented. Mean log_2 _ratios obtained during qRT-PCR experiments are plotted against the mean log_2 _ratios obtained with the microarrays. Numbers on the graph refer to the gene numbers in Table 4.

Seventeen genes, representing a wide range of log_2 _ratio values, were selected for transcript level analysis using qRT-PCR. Seven genes were overexpressed during iron restriction (*tonB1*, *hgbA*, *omp64*, *fetB2*, *apxIC*, PM0741, NMB1668); eight genes were repressed (*nrfA*, *nrfC*, *nfrE*, *ompW*, *dcuB2*, *dmsA*, *torA*, *ccmC*); two genes were not affected (*pedD*, *ap1465*). We also investigated the transcript level of the *exbB1*, *exbD1 *and *tbpA *genes, all known to be transcriptionally linked to *tonB1 *[17] and previously used as positive controls to assess iron restriction [12]. However, they were not present on the AppChip1 as this region of the genome was in one of the few unsequenced areas when the microarrrays were designed. In all cases, genes that had been identified as up- or down-regulated with the microarrays were confirmed by the qRT-PCR experiments. The *exbB1*, *exbD1 *and *tbpA *genes were also up-regulated. Genes not affected showed low level of variation during qRT-PCR analysis, and show good correlation with other results (Fig. 1). Overall, there was good correlation between the log_2 _ratios measured by microarray and log_2 _ratios from qRT-PCR data (R^2 ^= 0.87). The log_2 _ratios observed with qRT-PCR were usually superior to those observed with the microarray. This outcome has been observed before [25,26] and probably reflects the detection limit of microarrays as well as the complex normalization methods that are used prior to the analysis.

### Genes expressed differentially under iron restriction

To evaluate the effect of iron restriction on the porcine pathogen *A. pleuropneumoniae*, we performed microarray hybridization experiments. Given that iron plays a vital role in metabolic pathways through its presence in the structure of numerous enzymes [27] and its implication in the regulation of genes associated with virulence [28], we recorded important changes in the transcriptome of the bacteria under iron-restricted conditions. A total of 210 genes showed differential expression and the functional classification of these genes provides a significant overview of changes occurring in the bacteria. Numerous microarray studies have investigated effects of iron restriction in many different pathogens, including *E. coli *[29], *H. pylori *[30], *H. parasuis *[31], *N. gonorrhoeae *[25], *N. meningitidis *[32], as well as *Pasteurella multocida *[33], a well known animal pathogen closely related to *A. pleuropneumoniae*. Many genes that were identified as being iron-regulated in the *P. multocida *study were homologs of some genes that were also identified in our study (Table 3), thus emphasizing the importance of their regulation during iron restriction. A common feature in all these studies is the high induction of genes related to iron acquisition as the products of these genes are essential for survival of the bacteria.

#### (i) Down-regulated genes

**Figure 2:**
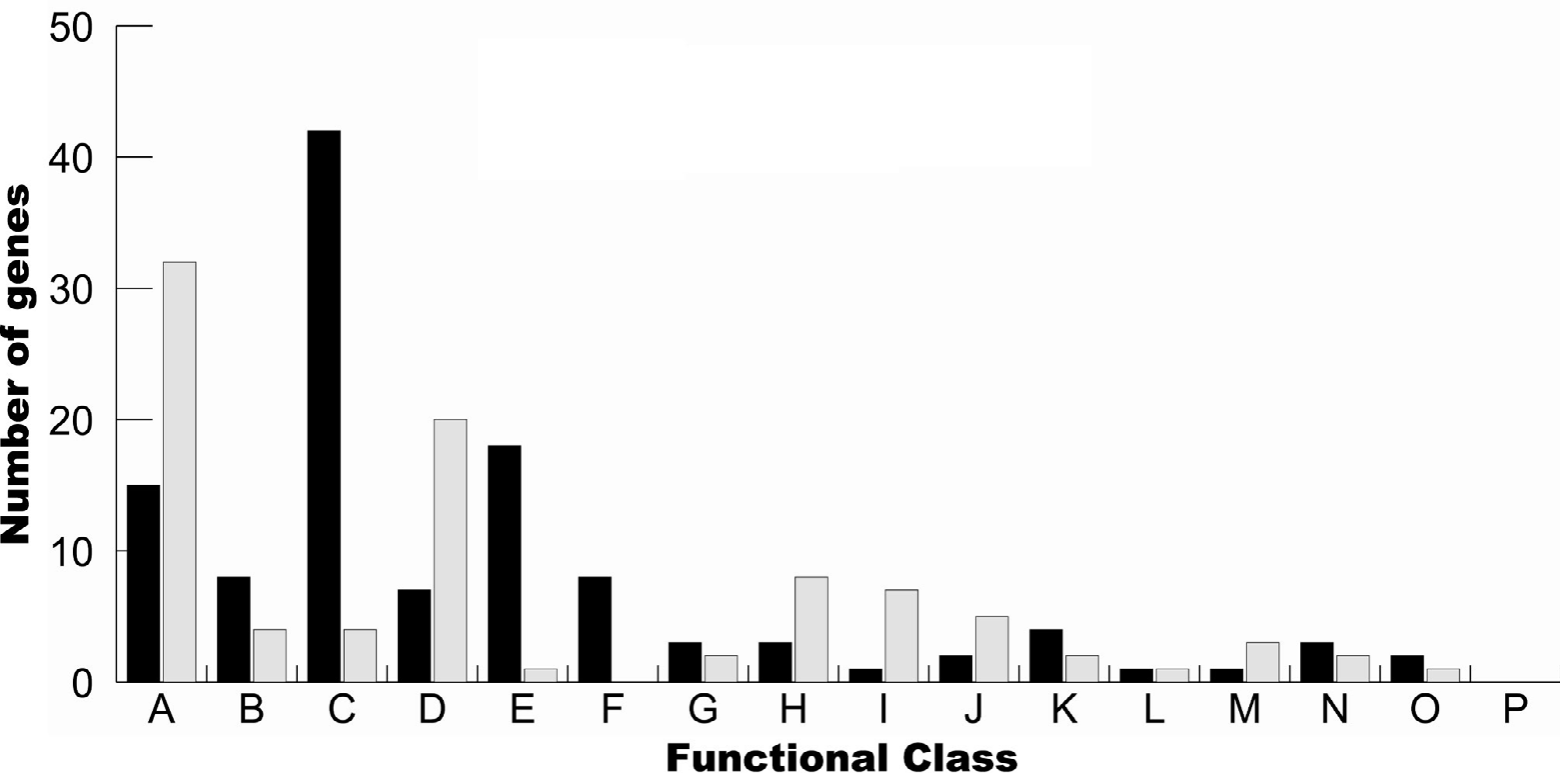
**Functional classification of the differentially expressed genes according to TIGRFAMs**. Black and grey bars respectively represent down-regulated and up-regulated genes. A: Hypothetical proteins/Unclassified/Unkown; B: Biosynthesis of cofactors, prosthetic groups and carriers; C: Energy Metabolism; D: Transport and binding proteins: cations and iron; E: Transport and binding proteins: others; F: Purines, pyrimidines, nucleosides and nucleotides; G: Regulatory functions; H: Protein fate; I: Protein synthesis; J: Cellular processes; K: Cell envelope; L: Fatty acids and phospholipids metabolism; M: Amino acids biosynthesis; N: DNA metabolism; O: Central intermediary metabolism; P: Mobile and extrachromosomal element functions.

Down-regulated genes (Fig. 2) mostly belong to the functional class termed "Energy Metabolism"; 42 of the 118 repressed genes (35%) belong to this group, and they are amongst the most highly repressed. Almost all these genes encode proteins with Fe-S clusters, that use heme molecules as cofactors, or that are activated by Fe^2+ ^or other divalent cations. These include genes coding for the different subunits of formate dehydrogenase (*bisC, hybA, fdhE*), fumarate reductase (*frdABC*), nitrate reductase (*nfrABC*), nitrate/trimethylamine oxidoreductase (TMAO) I and III (*torAC *and *torYZ*), dimethyl sulfoxide reductase (*dmsA*) and glycerol-3-phosphate dehydrogenase (*glpAC*). These enzymes as well as numerous others that encode either cytochrome components or functional partners (*cydAB, ccp*), cytochrome maturation proteins (*ccmCF, nfrE*) or iron-sulfur electron transport proteins (*napF, hyaA, ykgF*), are all implicated in the electron transport respiratory chain, either as electron donor or acceptor during aerobic and anaerobic respiration. Other genes in this category are involved in pathways of sugar metabolism such as fermentation (*pflB, adh2*), glycolysis or gluconeogenesis (*pgk, pfkA, gapA*) and the TCA cycle (*fumC, maeA*).

Many genes that are assigned to this category have been demonstrated or proposed to be members of the FNR regulon. The *E. coli *FNR transcriptional regulator is an oxygen-responsive activator implicated in the switch from aerobic to anaerobic metabolism in facultative anaerobes [34]. The oxygen-sensing domain of the FNR protein contains a Fe-S cluster, which is likely oxidized under aerobic conditions, thereby inactivating the FNR protein. Genes coding for fumarate and nitrate reductase are known to be influenced by FNR [35], as well as genes coding for anaerobic enzymes involved in the utilization of alternative terminal electron acceptors such as TMAO [36]. Sequence analysis in *H. influenzae *has identified conserved FNR binding motifs upstream of the *cydAB *genes [37]. These genes are usually considered to be up-regulated by the presence of the FNR protein, but FNR has also been implicated in the down-regulation of genes involved in aerobic respiration, such as genes coding for aerobic enzymes like NADH dehydrogenase and cytochrome oxidase [36]. In our study, although the *A. pleuropneumoniae *FNR homolog HlyX was observed to be up-regulated, all other putative members of the FNR regulon were shown to be down-regulated. In recent studies, genes *aspA*, coding for aspartate ammonia lyase, and *dmsA*, encoding a dimethyl sulfoxide reductase, were shown to be important for the virulence of *A. pleuropneumoniae *[38,39]. Both these genes, which are apparently under HlyX regulation [40], also showed down-regulation in our experiments. These results might indicate that another factor could interfere with HlyX regulation, or counter-balance the HlyX-inducing effect during iron restriction. The fact that most of the affected genes code for enzymes containing an Fe-S cluster in their structures or use iron as an activator [41] could explain this effect. Since studies have shown that the *A. pleuropneumoniae *FNR homolog may be involved in the activation of genes coding for virulence factors [42] and is essential for full virulence [40], the observed up-regulation of HlyX is not unexpected. Precise characterisation of the HlyX regulon in *A. pleuropneumoniae *will provide a better view of its role in pleuropneumonia. In *E. coli*, a second regulatory system, the ArcA/ArcB two component system, has also been shown to sense oxygen levels [43]. In our system, the *baeS *gene product, which has 51% identity with the *P. multocida Pm70 *ArcB protein, was also down-regulated, indicating that this system might be affected during iron restriction.

The overall picture of down-regulated genes shows that *A. pleuropneumoniae *has adopted strategies of economy for iron and energy. The principal components of the aerobic respiratory chain were all repressed, as well as key alternative final electron acceptors, probably since these processes implied extensive use of iron-containing enzymes. Genes involved in the synthesis of heme cofactors (*bioD1, hemA, hemC and hemN*) or quinones and menaquinones (*menA, menB *and *ispH*), which are important elements of the respiratory chain, showed down-regulation because lack of iron compromises these processes. Many components of the sugar phosphotransferase systems (PTSs) (*manX, hisS, ptsBHI*), which enable simultaneous transport and phosphorylation of sugars from phosphoenolpyruvate, as well as other genes involved in sugar transport (*dcuB1*,*dcuB2*,*rbsB*,*glpF*,*glpT*) were down-regulated under our experimental conditions. This outcome could hamper sugar uptake by the bacteria. Repression of the various PTSs might be caused by the repression of the *pfkA *gene, which codes for phosphofructokinase, a key enzyme in the pathway responsible for the conversion of glucose to phosphoenolpyruvate, and which serves as the primary source of phosphate for activation of PTSs [44]. The product of the *mlc *gene, which shows 70% homology with a probable *Haemophilus ducreyi *sugar metabolism repressor and which was also down-regulated, might be implicated in this down-regulation of PTSs. This repressor has been shown to repress the transcription of many PTSs, and is subject to a negative auto-regulation [44].

Considering these metabolic deficiencies, it is significant that some enzymes with ATPase activity as well as others involved in processes that are not of primary importance in adapting to iron restriction were down-regulated. As an example, four genes *purA, purT, pyrD, pyrG *for enzymes with ATPase activity that belong to the "Purine, Pyrimidines, Nucleosides and Nucleotides" functional class were down-regulated. Since bacteria are growing in a stressful environment and their metabolism seems highly compromised, expression of genes involved in the biosynthesis of molecules useful for replication is not essential.

#### (ii) Up-Regulated Genes

Many genes involved in cell metabolism were observed to be down-regulated by iron restriction, but cell metabolism was not highly represented in our set of up-regulated genes. Two genes showing high up-regulation during iron restriction were assigned to this category. The *lldD *gene showed a five-fold induction, and codes for L-lactate dehydrogenase, an enzyme required for conversion of lactic acid produced by fermentation to pyruvate. To compensate for defects of the respiratory chain, *A. pleuropneumoniae *might have started to rely on fermentation during iron restriction. The gene encoding the XylB xylose kinase involved in the degradation of xylose was also up-regulated. Considering that many PTSs were down-regulated, the use of this alternative sugar, for which PTS systems have seldom been implicated [45] may be reconciled. Several genes of the "Protein Fate" functional class also showed up-regulation. The two subunits of the Clp protease showed higher expression during iron restriction; this cytoplasmic protease is often involved in stress responses and protein quality control [46]. The genes *prlC *and *def*, encoding respectively an oligopeptidase and a peptide deformylase responsible for the hydrolysis of the N-formyl group of nascent polypeptide chains [47], were also up-regulated. This might indicate a higher turnover rate for native proteins requiring iron molecules in their structure, which might be unable to fold correctly in the absence of iron. The last gene of the "Protein Fate" functional class to be up-regulated was *mopB*, which codes for co-chaperonin GroES. This co-chaperonin, essential for full function of GroEL, facilitates non-native protein folding [48]. Again, the absence of iron might cause the accumulation of incorrectly folded native oligopeptide chains, thereby leading to higher expression of the GroES co-chaperonin.

The major response of *A. pleuropneumoniae *to iron restriction was the induction of genes involved in iron transport, probably to counter-balance effects of EDDHA. Most genes with known functions, identified as up-regulated during iron restriction, were shown to be involved in iron acquisition and transport. The *tonB1 *gene showed the highest level of up-regulation, and genes *exbB1*, *exbD1 *and *tbpA *which are transcriptionally linked to *tonB1 *were shown by qRT-PCR analysis to be also up-regulated. The *hgbA *gene was over-expressed, as well as the *hugZ *heme utilization protein which is located immediately upstream of *hgbA *[13]. Among other known *A. pleuropneumoniae *iron acquisition-related genes, *tonB2 *also showed up-regulation, while genes of the *fhu *operon did not show any significant change in expression, in agreement with previous work done in our laboratory; expression of *fhuA *is not regulated by iron [12].

**Figure 3:**
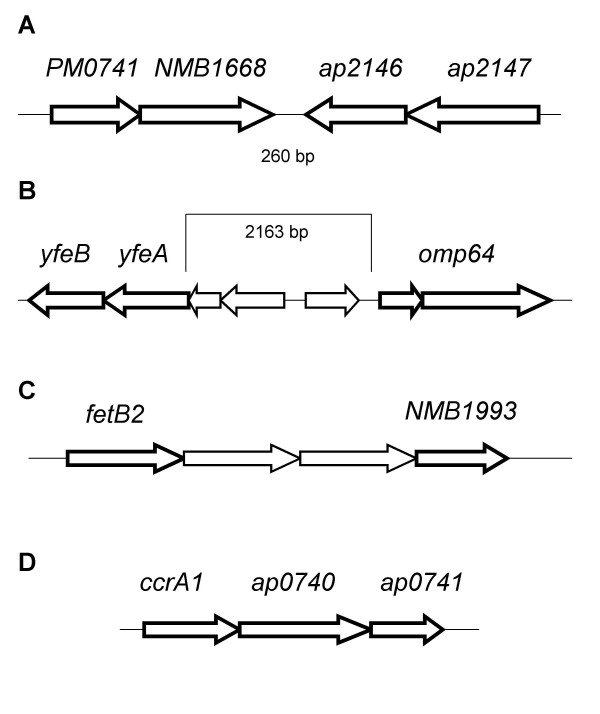
**Genetic organization of some gene clusters identified during this study**. a) Genomic region of the *A. pleuropneumoniae *5b strain L20 genome surrounding ORFs encoding for genes PM0741 and NMB1668. ORFs ap2146 and ap2147 are located 260 pb downstream of the NMB1668 ORF, and are transcribed in the opposite direction. b) *yfeAB *and *omp64 *genes are separated by three ORFs that did not show differential expression. c) Genetic organization of a possible operon coding for a putative enterobactin-type ABC transporter system. The two ORFs separating *fetB2 *and *NMB1993 *are the putative cytoplasmic components of this hypothetical system. d) The Fe^2+^/Pb^2+ ^high affinity permease locus.

Previously unreported iron acquisition systems were also revealed by our experiments. We identified a gene cluster, composed of ORFs PM0741 and NMB1668, showing 43% identity with the HmbR hemoglobin receptor from *N. meningitidis*. The HmbR receptor was shown in *N. meningitidis *to be important for survival in an infant rat infection model [49]. HmbR binds hemoglobin with high affinity, is able to strip heme from hemoglobin and then transport it to the periplasm. In *N. meningitidis*, HmbR is subject to phase variation via frameshift mutations [50], and about half of all clinical isolates express HmbR [51]. In *A. pleuropneumoniae*, the HgbA receptor has been shown to be responsible for iron acquisition from hemoglobin, and a mutant strain with an internal *hgbA *deletion could not grow in an iron-restricted medium supplemented with hemoglobin, albeit from different species [13]. Apparently HgbA is the sole hemoglobin receptor in *A. pleuropneumoniae *serotype 1, but it is not the sole hemoglobin binding protein that was identified in *A. pleuropneumoniae*. In the same study that lead to the identification of HgbA, a 75 kDa protein that could bind hemoglobin and hemin was also isolated [52]. The putative *A. pleuropneumoniae *HmbR has an estimated molecular weight of 76.7 kDa, and it is therefore tempting to speculate that those two proteins might share identity. In *N. meningitidis*, the *hmbR *gene is located downstream of *hemO *gene that codes for a heme oxygenase and that is considered essential for heme utilization by pathogenic *Neisseriae *[53]. No HemO homolog was found in the *A. pleuropneumonia*e genome, which might explain the apparent lack of iron acquisition from hemoglobin from other putative OM receptors than HgbA in *A. pleuropneumoniae*. Two other genes, located immediately downstream of the last NMB1668 ORF, and transcribed in the opposite direction, also showed up-regulation: ap2146 and ap2147; see Fig. 3. ORF ap2146 is predicted to code for the α subunit of a N-methylhydantoinase B/acetone carboxylase, while ORF ap2147 shares some region of homology with the periplasmic energy transducing protein TonB. Implication of the products of these ORFs in a potential iron-acquisition process involving the HmbR homolog remains to be assessed.

Our identification of a putative Yfe system was also of seminal interest. The Yfe system was first identified in *Y. pestis *and shown to allow chelated iron utilization in an *E. coli *mutant lacking enterobactin [54]. Two different operons encode the Yfe system, carrying genes *yfeABCD *and *yfeE *respectively; both operons were Fur-responsive. Later studies showed that the *yfeABCD *genes code for a periplasmic binding protein-dependent transport system belonging to the superfamily of ABC transporters [55], implicated in iron and manganese acquisition, and independent of TonB [56]. In *A. pleuropneumoniae*, homologs of components YfeABCD, showing respectively 63%, 76%, 75% and 66% homology with their counterparts in *P. multocida*, showed up-regulation during iron restriction, but were not present on the same operon. Gene *yfeB *can be found immediately downstream of the *yfeA *gene, in the same area as two other ORFs that were up-regulated during iron restriction and that could be implicated in iron acquisition. These ORFs, which are annotated as *omp64*, show good homology (32%) to the *Moraxella catarrhalis *CopB OM protein. Meanwhile, the *yfeCD *genes are located 160 kb downstream of the last *omp64 *ORF, and also show high homology with the corresponding *Y. pestis *Yfe proteins. The CopB protein has been implicated in iron acquisition from lactoferrin and transferrin; a mutant strain showed reduced ability to uptake iron from these proteins, with the more marked effect on transferrin-bound iron acquisition [57]. In *A. pleuropneumoniae*, proteins responsible for the utilization of transferrin-bound iron were first identified by affinity methods [58]. Later studies showed that the *tbpAB *genes from *A. pleuropneumoniae *are transcriptionally linked to genes *tonB1*, *exbB1 *and *exbD1*, and these *exb *genes are essential for iron acquisition from transferrin [17]. It was also shown that both *tbp *genes are essential for iron acquisition from transferrin [59], and another recent study showed that a *tonB1 *mutant cannot use porcine transferrin, but is not attenuated *in vivo *[18]. As with the *A. pleuropneumoniae *HmbR homolog, it would be interesting to examine the presence of the CopB homolog *in vitro *and *in vivo*, and possible effects of mutations in this gene. Although the best amino acid homology was with the CopB protein, the overall alignment of the *A. pleuropneumoniae omp64 *gene product with the *M. catharralis *CopB protein is not strong, implying that Omp64 might have a role to play in iron acquisition, but its target might not be transferrin or lactoferrin. Another ORF (ap1453) also annotated *omp64 *showed up-regulation in our experiments. This second Omp64 also shows homology with the CopB OM protein (57%), but the overall alignment with CopB seems superior to that of the other Omp64 (ap0300 – ap0301). The ap1453 Omp64 also shows homology with the *H. influenzae *heme-hemopexin utilization protein C (41%). We hypothesize that the YfeABCD-Omp64 (ap0300-ap0301) proteins are components of a new iron acquisition system in *A. pleuropneumoniae *that are located in the OM (Omp64) and in the CM (YfeBCD), as determined by the PSORT algorithm [60]. The exact location of YfeA could not be determined precisely, but it is predicted not to be cytoplasmic.

Another cluster of genes was particularly interesting with regards to iron acquisition. Two ORFs, annotated *fetB2*, seem to encode a unique protein presenting similarities with members of the TroA superfamily of periplasmic metal binding proteins. Sequence analysis reveals homologies with other known or putative periplasmic binding proteins, some of which are involved in iron transport. Downstream, three putative genes appear to code for the components of an ABC transport system. One gene of this putative ABC transport system was also up-regulated: the *NMB1993 *gene coding for a putative ATPase component. These components show homology with the Ceu system (*Campylobacter *Enterochelin Utilization), and prediction of localization with PSORT indicates that *fetB2 *localizes to the periplasm, *NMB1993 *to the CM and/or cytoplasm; the two other components, which are not over-expressed in our system, were predicted to be in the CM. We demonstrated [20,61] that *A. pleuropneumoniae *uses different exogenous siderophores, including a catechol-type siderophore like enterochelin. Up to now, in *A. pleuropneumoniae*, the only identified siderophore OM receptor is FhuA, specific for ferric hydroxamates [11]. It is premature to conclude that the *fetB2 *and *NMB1993 *genes are part of this unidentified catechol-type siderophore acquisition system.

Three other up-regulated ORFs were identified as having a putative role in iron acquisition. ORFs *ccrA1*, ap0740 and ap0741 were classified as proteins of unknown function, but share homologies respectively with a family of periplasmic lipoproteins involved in iron transport, a family of iron-dependent peroxidase and a family of high affinity Fe^2+^/Pb^2+ ^permease. Since no clear homology with any known or characterized protein was established, hypotheses concerning their function and roles in iron acquisition have to be formulated with great care. Recently, Cowart showed [62] that bacterial reductases, by changing the state of free iron from Fe^3+ ^to Fe^2+^, could play a major role in iron acquisition. The presence of a possible Fe^2+ ^permease could indicate the existence of such a mechanism in *A. pleuropneumoniae*.

Considering that iron restriction conditions are encountered *in vivo*, we further examined the expression of known or putative virulence factors of *A. pleuropneumoniae *under such conditions. Aside from different iron acquisition systems, the Apx toxins are often regarded as essential for virulence of *A. pleuropneumoniae*. The Apx toxins are members of the RTX (Repeat in Toxin) family, and the genetic organization of the genes that are essential for the synthesis and secretion of the toxin generally follows the same order: *apxC*, *apxA*, *apxB *and *apxD*, which code respectively for the pretoxin activator, the pretoxin structure and the secretion apparatus [63]. These genes can be transcribed from two different transcripts: a major 3.5 kb transcript containing genes *apxICA*, and a minor 7.5 kb transcript with genes *apxICABD *[64]. During iron restriction, the first gene of the *apxI *operon, *apxIC*, showed slight up-regulation, but the three other genes were not over-expressed. The *A. pleuropneumoniae *strain used in this study possesses genes coding for the ApxI, II and IV toxins. Very little is known about the transcriptional regulation of the *apxI *operon, but it has been shown to be at least regulated by the combined activity of the Fur protein and calcium [21]. Under high calcium concentration, Fur seemed to act as an activator of the *apxI *operon, while it seemed to act as a repressor under low calcium concentration. Under our experimental conditions, it seems that Fur acts as a repressor since the *apxIC *gene was identified as being slightly up-regulated during iron-restriction, i.e. in the absence of Fur. The fact that it was the only gene of the *apxI *operon to show significant up-regulation might reflect the stringency of our analysis, but might also point towards the existence of fine post-transcriptional tuning of the *apxI *operon. The existence of such mechanisms of regulation could also explain why *apxIA *does not seem to be up-regulated, even though it is located on the same transcript as *apxIC*.

The *A. pleuropneumoniae ureC *has been implicated as a possible virulence factor, with a putative role in persistence of bacteria *in vivo *[65]. In our study, the *ureC *gene was identified as being down-regulated during iron restriction. Since it was shown that this gene might have more effect in the late stage of the disease, it seems clear that other *in vivo *factors, as with *hlyX*, may influence the regulation of the *ureC *gene.

Three ORFs which had approximately two-fold induction during iron deficiency also warrant attention. The Ssa1 protein (Serotype 1-Specific Antigen) was first identified in *Mannheimia haemolytica *and was associated with the serotype switch occurring in the upper respiratory tract of bovines following stressful events, potentially leading to development of disease [66]. The protein was shown to localize to the OM [67]. Sequence homology research on the *A. pleuropneumoniae *Ssa1 protein led to identification of an autotransporter domain at the C-terminal extremity of the protein; the *A. pleuropneumoniae *Ssa1 was also classified in the family of subtilisin-like serine proteases, although no experimental evidence of this activity could be found. Recently, autotransporter proteins such as Ig proteases have been implicated in virulence [68]. Autotransporter proteins belonging to the serine protease family have been identified in various Gram-negative bacteria. *H. influenzae*, a close relative of *A. pleuropneumoniae*, possesses at least two: an IgA1 protease and the Hap protein which has been shown to be involved in adhesion. Little is known about the Ssa1 protease but its implication in virulence in *M. haemolytica *suggests that it could play a similar role in *A. pleuropneumoniae*.

Genes *ftsK *and *ftsA*, essential in the first steps of cell division, also showed higher expression during iron restriction. Considering that some genes involved in the "Purine, Pyrimidines, Nucleosides and Nucleotides" were down-regulated, this result was unexpected. However, it has been shown that the transcription of the *ftsZ *gene is subjected to regulation by antisense transcription of a 490-bp segment spanning the junction between the *ftsA *and *ftsZ *genes [69], which could probably explain the apparent overproduction of the *ftsA *mRNA. As for *ftsK*, although the protein is implicated in cell division, other functions have been suggested for this protein [70], and the observed up-regulation might not be linked with cell division.

## Conclusion

In summary, the evaluation of differential gene expression in *A. pleuropneumoniae *during growth in an iron-restricted medium enabled us to gain a better understanding of the metabolic changes occurring in response to this stress. Transcript profiling using DNA microarrays is a powerful tool to determine the exact composition of the bacterial transcriptome in defined conditions, therefore leading to the putative identification of components that are essential during these conditions. It can also help identify components which are likely to be expressed during the infection process in the host, and that might be interesting targets for vaccines.

In the course of our study, many new potential iron acquisition systems were highlighted. Clearly, iron acquisition in *A. pleuropneumoniae *might rely on more systems that what was previously thought, and further studies will be necessary to evaluate the impact of these systems during the course of infection by *A. pleuropneumonia*.

## Methods

### Bacterial strains and growth conditions

*Actinobacillus pleuropneumoniae *serotype 1 strain S4074 was routinely grown on BHI medium supplemented with either 15 μg/ml (agar) or 5 μg/ml (broth) of NAD. For the microarray experiments, two flasks of BHI broth were inoculated with 500 μl of an overnight culture of *A. pleuropneumoniae *serotype 1 strain S4074 and grown at 37°C in an orbital shaker until an optical density of 0.1 was reached. To initiate iron restriction in one of the two cultures, EDDHA was added to a final concentration of 50 μg/ml. In the iron supplementation experiments, FeCl_3 _was added to the iron depleted culture 5 min. after the addition of EDDHA to a final concentration of 50 μg/ml. The cultures were then re-incubated until they reached a final optical density of 0.3.

### RNA extraction

RNA was harvested from cells at an optical density of 0.3. Ice-cold RNA degradation stop solution (95% ethanol, 5% buffer-saturated phenol), shown to effectively prevent RNA degradation and therefore preserve the integrity of the transcriptome [71], was added to the bacterial culture at a ratio of 1:10 (vol/vol). The sample was mixed by inversion, incubated on ice for 5 min, and then spun at 5000 g for 10 min to pellet the cells. Bacterial RNA isolation was then carried out using the QIAGEN RNeasy MiniKit. During the extraction, samples were subjected to an on-column DNase treatment, as suggested by the manufacturer. The RNA concentration, quality and integrity were assessed spectrophotometrically and by gel analyses.

### Construction of the *A. pleuropneumoniae *5b strain L20 microarray (AppChip1)

The draft genome sequence of *A. pleuropneumoniae *serotype 5b strain L20 [GenBank: CP000569] was used as a source of the genes used in this study. ORFs were identified using the Glimmer software package [72], and used to search for homologs among the bacterial gene subset of Genbank [73] using the BLASTP program [74]. PCR primers were designed for each of the 2025 ORFs of the genome of *A. pleuropneumoniae *using the Primer3 program [75] controlled by an automated script as described previously [76]. Primer-selection parameters were standardized and included a similar predicted melting temperature (60 ± 3°C), uniform length (25 nt), and a minimum amplicon size of 160 bp. Generation of PCR amplicons and fabrication of DNA microarrays were as described [76]. Details on the construction of this microarray (AppChip1) are available on the Institute for Biological Sciences website [77].

### Microarray hybridizations

cDNA synthesis and microarray hybridizations were performed as described [78]. Briefly, equal amounts (15 μg) of test RNA and control RNA were used to set up a standard reverse transcription reaction using random octamers (BioCorp), SuperScript II (Invitrogen) and aminoallyl-dUTP (Sigma), and the resulting cDNA was indirectly labelled using a monofunctional NHS-ester Cy3 or Cy5 dye (Amersham). The labelling efficiency was assessed spectrophotometrically. Labelled samples were then combined and added to the *A. pleuropneumoniae *5b strain L20 microarray. Nine hybridizations were performed for the iron-restriction experiments, including three pairs of microarrays for which Cy3 and Cy5 dyes were swapped, while 4 hybridizations were conducted for the iron supplementation experiments. Data were submitted to the Gene Expression Omnibus [79] [GEO:GSE4674 and GSE6366]. All slides were scanned using a Perkin-Elmer ScanArray Express scanner.

### Microarray analysis and bioinformatics

The TM4 suite of software from The Institute of Genomic Research was used for the whole microarray analysis [80]. First, raw data were generated using SpotFinder v.3.0.0 beta. The integrated intensities of each spot, equivalent to the sum of intensities of all unsaturated pixels in a spot, were quantified and the integrated intensity of the local background was subtracted for each spot. The same operation was performed with the median spot intensities. The spot detection threshold was set so that spots for which the integrated intensity was less than one standard deviation above the background median intensity were set to zero. Raw spot data were converted from integrated intensities to median spot intensities using TIGR's Express Converter software, the latter being less influenced by outlier values than integrated intensities.

Data were normalized with TIGR's MIDAS software tool using locally weighted linear regression (lowess) [81-83]. Spots with median intensities lower than 1000 were removed from the normalized data set. Intensities for duplicate spots were merged to generate the final normalized data set, subsequently analyzed using TIGR's TMEV microarray analysis tool. The Significance Analysis of Microarray (SAM) algorithm [84], which is implemented in TMEV, was used to generate a list of differentially expressed genes. During SAM analysis, a false discovery rate of 3.22% was estimated for the iron-depleted *versus *BHI broth experiment, while a FDR of 2.51% was estimated for the iron-supplemented *versus *BHI broth experiment; this value estimates the proportion of genes likely to have been identified by chance. Functional classification of these genes was conducted using TIGR's Comprehensive Microbial Resource (CMR) [85]. Proteins were assigned to their corresponding pathways using the MetaCyc Metabolic Pathway Database [41]. Homologies were assessed using Blast tools [86] hosted on the NCBI and TIGR servers. Additional subcellular localization was determined with PSORTb [60]. Protein sequence alignments were performed using the ClustalW multiple sequence alignment algorithm [87].

### Real-Time quantitative RT-PCR

Microarray results were verified by real-time quantitative RT-PCR (qRT-PCR), using the QuantiTect^® ^SYBR^® ^Green RT-PCR Kit (Qiagen). Reactions were performed with a 16-place Cepheid Smart Cycler^® ^System in a total volume of 25 μl. Oligonucleotide primers (Table 4) were designed using Primer3 software [75]. To ensure that amplification with these primers resulted in single amplicon of the anticipated size, they were PCR tested before proceeding to qRT-PCR analysis. Primer pairs which amplified fragments of 195 to 205 bp with a melting temperature of 60°C were selected. Seventeen genes (7 up-regulated, 8 down-regulated, 2 non-significant) were selected for analysis. Relative expression of each gene as determined by qRT-PCR was normalized to that of the *ackA *gene which showed a stable level of expression throughout the different microarray experiments (data not shown). Prior to the qRT-PCR, the RNA samples were subjected to a DNase treatment with TURBO DNase (Ambion, Austin, TX) to avoid DNA contamination in the samples. Quantitative measures were obtained using the 2−ΔΔCT
 MathType@MTEF@5@5@+=feaafiart1ev1aaatCvAUfKttLearuWrP9MDH5MBPbIqV92AaeXatLxBI9gBaebbnrfifHhDYfgasaacH8akY=wiFfYdH8Gipec8Eeeu0xXdbba9frFj0=OqFfea0dXdd9vqai=hGuQ8kuc9pgc9s8qqaq=dirpe0xb9q8qiLsFr0=vr0=vr0dc8meaabaqaciaacaGaaeqabaqabeGadaaakeaacqaIYaGmdaahaaWcbeqaaiabgkHiTiabfs5aejabfs5aejabboeadnaaBaaameaacqqGubavaeqaaaaaaaa@33ED@ method [88].

## List of abbreviations

BHI : Brain Heart Infusion, CGH : Comparative Genomic Hybridization, EDDHA : ethylenediamine dihydroxyphenyl acetic acid, Fe-S : iron-sulfur, NAD : Nicotinamide Adenosine Dinucleotide, Ig : Immunoglobulin, ORF : Open Reading Frame, TCA : Tricarboxylic Acid.

## Authors' contributions

VD designed the transcript profiling experiments, carried out downstream data analysis, and drafted the manuscript. JHEN designed the AppChip1 and helped with the downstream data analysis. JWC and JH participated in the study design and revised the manuscript. MJ participated in the conception and supervised the design of the study and revised the manuscript. All authors read and approved the final manuscript.

## Figures and Tables

**Table 1:** *A. pleuropneumoniae *genes which are down-regulated during iron restriction

**Gene ID**	**Gene**	**Description**	**Fold Change**
*Hypothetical/Unclassified/Unknown*			
ap0497	*engA*	putative GTP binding protein	-2.27
ap0491	*glnE*	Unknown	-1.98
ap1365	*srmB*	uncharacterized conserved protein	-1.85
ap1538	*traC*	conserved hypothetical protein	-1.72
ap0677	*nfnB*	putative nitroreductase, FMN-dependent	-1.70
ap1779	*mscL*	conserved hypothetical protein	-1.69
ap0802	*dxr*	conserved hypothetical protein, distant homolog of PhoU	-1.58
ap0787	*cdsA*	putative transcriptional regulator	-1.54
ap0685	*mlc*	protein of unknown function	-1.53
ap1297^+^	*sspA*	predicted iron-dependent peroxidase	-1.53
ap0973	*abgB*	possible metal dependent peptidase, unclassified	-1.48
ap1405	*nth*	possible sodium/sulphate transporter	-1.41
ap1725	*mviN*	uncharacterized membrane protein, putative virulence factor	-1.38
ap0622	*aroC*	*flp *operon protein C	-1.28
ap0989	*fstX*	conserved hypothetical protein	-1.27
			
*Biosynthesis of cofactors*			
ap0684	*bioD1*	probable dethiobiotin synthetase	-3.49
ap1624	*menA*	1,4-dihydroxy-2-naphthoateoctaphenyltransferase	-1.57
ap1131	*hemC*	porphobilinogen deaminase	-1.47
ap0447	*hemA*	glutamyl-tRNA reductase	-1.40
ap1080	*hemN*	oxygen-independent corproporphyrinogen III oxydase	-1.40
ap2005	*menB*	naphthoate synthase	-1.39
ap1684	*ispH*	hydroxymethylbutenyl pyrophosphate reductase	-1.37
ap2023	*-*	4-hydroxybenzoate synthetase	-1.31
			
*Energy Metabolism*			
ap0108^+^	*nrfA*	nitrate reductase cytochrome c552	-10.48
ap1694^+^	*frdA*	fumarate reductase flavoprotein subunit	-9.20
ap1693^+^	*frdB*	fumarate reductase iron-sulfur protein	-7.86
ap1536	*ccp*	cytochrome C peroxidase	-6.61
ap0764^+^	*torY*	nitrate/TMAO reducatse, tetraheme cytochrome C subunit	-6.27
ap0996^+^	*bisC*	nitrate-inducible formate dehydrogenase-N α subunit	-5.68
ap0997^+^	*bisC*	nitrate-inducible formate dehydrogenase-N α subunit	-5.40
ap0762^+^	*torZ*	trimethylamine-N-oxide reductase 2	-5.23
ap0998^+^	*hybA*	formate dehydrogenase β subunit	-5.23
ap0498^+^	*ykgF*	putative Fe-S electron transport protein	-4.78
ap1692^+^	*frdC*	fumarate reductase 15 kD hydrophobic protein	-4.67
ap1937	*fumC*	fumarate hydratase class II	-4.45
ap0499^+^	*ykgE*	conserved putative dehydrogenase, Fe-S oxidoreductase	-4.38
ap1132^+^	*adh2*	alcool dehydrogenase 2 dehydrogenase	-3.36
ap1163^+^	*pflB*	formate acetyltransferase	-3.01
ap1221	*aspA*	aspartate ammonia-lyase	-2.78
ap1848^+^	*dmsA*	dimethyl sulfoxyde reductase	-2.73
ap1222	*aspA*	aspartate ammonia-lyase	-2.69
ap0110^+^	*nrfC*	nitrate reductase, Fe-S protein	-2.63
ap0380	*glgB*	1,4-α-glucan branching enzyme	-2.55
ap0414	*glpK*	putative glycerol kinase	-2.29
ap0109^+^	*nrfB*	nitrate reductase, cytochrome C-type protein	-2.25
ap1255	*pfkA*	Phosphofructokinase	-2.20
ap1486^+^	*hyaA*	Ni-Fe hydrogenase I small subunit	-2.09
ap1525^+^	*ccmF*	cytochrome C-type biogenesis protein	-1.98
ap1181^+^	*nfrE*	cytochrome c-type biogenesis protein	-1.88
ap0418^+^	*glpA*	anaerobic glycerol-3-phosphate dehydrogenase, subunit A	-1.76
ap0958^+^	*sdaA*	L-serine dehydratase	-1.75
ap0420^+^	*glpC*	anaerobic glycerol-3-phosphate dehydrogenase, subunit C	-1.72
ap1979	*torA*	trimethylamine oxydoreductase precursor	-1.70
ap1528^+^	*ccmC*	cytochrome C-type biogenesis protein	-1.69
ap1000^+^	*fdhE*	formate dehydrogenase formation protein	-1.62
ap0328^+^	*cydB*	cytochrome D ubiquinol oxidase subunit II	-1.61
ap1588^+^	*napF*	ferredoxin-type protein	-1.55
ap1402	*pgk*	phosphoglycerate kinase	-1.55
ap1585^+^	*torC*	nitrate/TMAO reductase, tetraheme cytochrome C subunit	-1.53
ap0089	*dAK1*	dihydroxyacetone kinase	-1.51
ap0541	*maeA*	malate oxydoreductase	-1.46
ap0326^+^	*cydA*	cytochrome D ubiquinol oxidase subunit I	-1.45
ap0484	*gapA*	glyceraldehydes-3-phosphate dehydrogenase	-1.35
ap1822	*atpH*	ATP synthase δ chain	-1.28
ap1116	*galK*	Galactokinase	-1.26
			
*Transport and binding proteins: cations and iron*			
ap0169^+^	*aopA*	NADH-ubiquinone oxidoreductase, Na^+^-translocating A subunit (nqrA)	-2.38
ap0354	*nhaB*	Na^+^/H^+ ^antiporter protein	-2.14
ap0170^+^	*nqrB*	NADH degydrogenase, Na^+^-translocating B subunit	-2.09
ap0172^+^	*nqrD*	NADH-ubiquinone oxidoreductase, Na^+^-translocating D subunit	-2.05
ap0171^+^	*nqrC*	NADH-ubiquinone oxidoreductase, Na^+^-translocating C subunit	-2.02
ap1972	*nadR*	putative periplasmic binding protein, ABC metal ion uptake	-1.61
ap0173^+^	*nqrE*	NADH-ubiquinone oxidoreductase, Na^+^-translocating E subunit	-1.52
			
*Transport and binding proteins: others*			
ap1470	*dcuB2*	anaerobic C4-dicarboxylate membrane transporter	-5.81
ap0416	*glpT*	glycerol-3-phosphate transporter	-3.71
ap1835	*manX*	PTS system enzyme IIAB, mannose specific	-2.28
ap1548	*mMT1*	PTS system mannose-specific EII AB component	-1.83
ap1473	*ptsB*	PTS system, sucrose-specific IIBC component,	-1.67
ap1477	*ptsH*	PTS system phosphocarrier protein HPr	-1.65
ap1620	*glpF*	glycerol uptake facilitator	-1.56
ap1164	*focA*	probable formate transporter	-1.54
ap0924	*cydC*	ABC transporter involved in cytochrome bd biosynthesis	-1.51
ap1833	*hisS*	PTS system component IID, mannose specific	-1.48
ap1580	*rbsB*	galactose ABC transporter, periplasmic binding protein	-1.48
ap0886	*sapC*	peptide transport system permease protein	-1.39
ap1698	*dcuB1*	anaerobic C4-dicarboxylate transporter	-1.38
ap2065	*mscS*	small-conductance mechanosensitive channel	-1.37
ap1367	*PM0514*	permease of unknown function	-1.34
ap1478	*ptsI*	phosphoenolpuruvate PTS system enzyme I	-1.32
ap1463	*proP*	permease of the major facilitator superfamily	-1.32
ap1507	*artQ*	arginine transport system permease protein	-1.22
			
*Purines, pyrimidines, nucleosides and nucleotides*			
ap2022	*udp*	uridine phosphorylase	-2.21
ap1237	*purT*	phorphoribosyglycinamide formyltransferase 2	-1.67
ap0154	*pyrG*	CTP synthase	-1.54
ap1922	*cdpC*	2',3'-cyclic-nucleotide 2'-phosphodiesterase	-1.46
ap0862	*pyrD*	dihydroorotate dehydrogenase	-1.42
ap1204	*purA*	adenylosuccinate synthetase	-1.37
ap0863	*prsA*	ribose-phosphate pyrophosphokinase	-1.34
ap0729	*purE*	phosphoribosylaminoimidazole carboxylase catalytic subunit	-1.33
			
*Regulatory functions*			
ap1392	*ansB*	probable carbon starvation protein A, membrane bound	-2.59
ap1803	*glpR*	transcriptional regulator of sugars metabolism	-1.51
ap1048	*baeS*	sensory transduction histidine kinase	-1.36
			
*Protein fate*			
ap1485^+^	*hypF*	Ni-Fe hydrogenase maturation protein	-2.39
ap2081	*lgt*	prolipoprotein diacylglyceryl transferase	-1.58
ap0428	*pepB*	peptidase B	-1.38
			
*Protein synthesis*			
ap0241	*thrS*	threonyl-tRNA synthetase	-1.40
			
*Cellular processes*			
ap0725	*uspA*	universal stress protein A	-1.59
ap0333	*tolB*	colicin tolerance protein	-1.29
			
*Cell envelope*			
ap1215	*ompW*	outer membrane protein W	-10.00
ap1156	*rplK*	COG5039: exopolysaccharide biosynthesis protein	-1.32
ap0021	*HI1139*	UDP-N-acetylmuramate-alanine ligase (murC)	-1.23
ap1154	*ushA*	glycosyltransferase involved in LPS biosynthesis	-1.19
			
*Fatty acids and phospholipids metabolism*			
ap2049	*accC*	biotin carboxylase	-1.24
*Amino acids biosynthesis*			
ap0351	*OB1054*	putative methionine synthase	-1.55
			
*DNA metabolism*			
ap1336	*-*	putative *hsdR*, type 1 site-specific restriction-modification system, R subunit	-1.54
ap0703	*alxA-hsdM*	type I restriction-modification system methylation subunit	-1.41
ap1247	*recQ*	ATP-dependent DNA helicase	-1.21
			
*Central intermediary metabolism*			
ap1787	*ureC*	urease α subunit	-1.45
ap1785	*ureE*	metallochaperone for urease	-1.22

^+ ^Genes coding for iron-containing proteins or proteins using Fe^2+ ^as a cofactor.

**Table 2:** *A. pleuropneumoniae *genes which are up-regulated during iron restriction

**Gene ID**	**Gene**	**Description**	**Fold Change**
*Hypothetical/Unclassified/Unknown*			
ap2147^+^	*-*	possible N-methylhydantoinase B/acetone carboxylase, α subunit	6.22
ap0740	*-*	predicted iron-dependent peroxidase	3.56
ap2196	*PM1515*	protein of unknown function	3.40
ap0741^+^	*-*	predicted high-affinity Fe^2+^/Pb^2+ ^permease	3.06
ap2146	*-*	possible N-methylhydantoinase B/acetone carboxylase, α subunit	2.88
ap0739^+^	*ccrA1*	predicted periplasmic protein involved in iron transport	2.69
ap2014	*rpmJ1*	conserved hypothetical protein	2.01
ap0286	*nagB*	conserved hypothetical protein	1.85
ap1686	*araJ*	conserved hypothetical protein	1.84
ap2182	*rpsU*	conserved hypothetical protein	1.83
ap2207	*PM1452*	protein of unknown function	1.63
ap0035	*-*	hypothetical protein	1.62
ap1927	*-*	outer membrane lipoprotein A	1.59
ap1436	*NMA1782*	conserved hypothetical protein	1.57
ap0755	*aroA*	conserved hypothetical protein	1.55
ap0874	*-*	hypothetical protein	1.54
ap0143	*rplI*	HIT-like protein	1.51
ap0056	*typA*	predicted membrane GTPase involved in stress response	1.51
ap1364	*add*	conserved hypothetical protein	1.49
ap1252	*icc*	conserved putative lipoprotein	1.45
ap0371	*yrbK*	conserved hypothetical protein	1.43
ap0478	*HI0719*	conserved hypothetical protein	1.43
ap1444	*fimD*	conserved hypothetical protein	1.43
ap1598	*slyD*	hypothetical protein	1.41
ap0907	*HI1265*	conserved hypothetical protein	1.41
ap0375	*firA*	conserved hypothetical protein	1.39
ap0079	*comF*	conserved glutaredoxin-like protein	1.37
ap0329	*mlc*	conserved hypothetical protein	1.36
ap1664	*HI1720*	conserved hypothetical protein	1.34
ap0059	*dnaQ*	uncharacterized stress-induced protein	1.30
ap1172	*PM1281*	predicted permease	1.30
ap0324	*ureF*	conserved hypothetical protein	1.16
			
*Biosynthesis of cofactors*			
ap0423	*ribB*	riboflavin synthase α subunit	1.59
ap0422	*ribG*	riboflavin-specific deaminase	1.43
ap0947	*licA*	putative oxygen-independent coproporphyrinogen III oxidase (HemN)	1.37
ap1036	*fdx2*	ferredoxin	1.35
			
*Energy metabolism*			
ap2032	*lldD*	l-lactate dehydrogenase	4.98
ap1733	*xylB*	probable L-xylulose kinase (L-xylulokinase)	3.04
ap1424	*ndh*	NADH dehydrogenase	1.86
ap1363	*fldA*	flavodoxin	1.61
			
*Transport and binding proteins: cations and iron*			
ap1740^+^	*tonB1*	energy transducing protein	8.71
ap2142^+^	*PM0741*	outer membrane protein, Fe transport, hemoglobin	6.15
ap1175^+^	*hgbA*	hemoglobin-binding protein precursor	5.89
ap1176^+^	*hgbA*	hemoglobin-binding protein precursor	5.45
ap2144^+^	*NMB1668*	hemoglobin receptor	4.78
ap1177^+^	*hugZ*	heme utilization protein	4.14
ap0295^+^	*yfeA*	iron (chelated) ABC transporter, periplasmic-binding protein	3.88
ap2143^+^	*PM0741*	outer membrane protein, Fe transport, haemoglobin	3.66
ap0296^+^	*yfeA*	iron (chelated) ABC transporter, periplasmic-binding protein	3.55
ap1453^+^	*omp64*	outer membrane protein, TonB dependent receptor	3.09
ap0294^+^	*yfeB*	iron (chelated) transporter, ATP-binding protein	2.98
ap2145^+^	*NMB1668*	hemoglobin receptor	2.85
ap0300^+^	*omp64*	outer membrane protein, TonB dependent receptor	2.07
ap0301^+^	*omp64*	outer membrane protein, TonB dependent receptor	1.93
ap0797^+^	*fetB2*	putative ferric enterobactin transporter binding protein	1.76
ap0796^+^	*fetB2*	putative ferric enterobactin transporter binding protein	1.60
ap0082^+^	*tonB2*	energy transducing protein	1.57
ap0801^+^	*NMB1993*	iron(III) ABC transporter, ATP-binding protein	1.49
ap0144^+^	*yfeD*	iron (chelated) transport system, membrane protein	1.47
ap0145^+^	*yfeC*	iron (chelated) transport system, membrane protein	1.38
			
*Transport and binding proteins: others*			
ap1437	*NMA0994*	putative periplasmic protein	1.58
			
*Regulatory functions*			
ap0726	*hlyX*	FNR-like transcriptional regulator	2.63
ap0652	*HI0893*	transcriptionnal repressor Bm3R1	1.24
			
*Protein fate*			
ap0399	*ssa1*	subtilisin-like serine protease	2.36
ap0400	*ssa1*	subtilisin-like serine protease	2.22
ap0401	*ssa1*	subtilisin-like serine protease	2.01
ap1887	*def*	peptide deformylase	1.64
ap1432	*clpP*	ATP-dependent Clp protease	1.54
ap1160	*prlC*	oligopeptidase A	1.39
ap1134	*mopB*	heat-shock 10 protein GroES	1.36
ap1431	*clpX*	ATP-dependent Clp protease, ATP-binding ClpX subunit	1.20
			
*Protein synthesis*			
ap0337	*tdk*	probable tRNA-dihydrouridine synthase C	1.95
ap1295	*potD1*	probable pseudo-uridine synthase	1.35
ap1895	*rplK*	50S ribosomal protein L11	1.32
ap1305	*rplI*	50S ribosomal protein L9	1.31
ap1253	*rluD*	pseudo-uridine synthase	1.24
ap0245	*infC*	translation initiation factor IF-3	1.23
ap1666	*valS*	valyl-tRNA synthetase	1.22
			
*Cellular processes*			
ap0168	*napC*	transformation locus protein OrfG	1.62
ap1505	*HI1275*	tellurite resistance protein TehB	1.61
ap1606	*apxIC*	RTX-1 toxin determinant	1.57
ap0688	*ftsK*	cell division protein FtsK	1.27
ap0025	*ftsA*	cell division protein FtsA	1.22
			
*Cell envelope*			
ap0486	*mreB*	similar to rod shape-determining protein MreB	1.33
ap0507	*lapB*	putative membrane protein, virK family member	1.30
			
*Fatty acids and phospholipids metabolism*			
ap1649	*accA*	acetyl-CoA carboxylase carboxyl transferase α subunit	1.38
			
*Amino acids biosynthesis*			
ap2037	*ilvC*	ketol-acid reductoisomerase	2.38
ap1566	*gshA*	putative gluthatione biosynthesis bifunctionnal protein	1.51
ap0466	*argG*	argininosuccinate synthetase	1.24
			
*DNA metabolism*			
ap2148	*mutL*	DNA mismatch repair protein MutL	1.34
ap2202	*srmB*	ATP-dependent RNA helicase	1.19
			
*Central intermediary metabolism*			
ap1688	*HI0111*	gluthatione transferase	1.24

^+ ^Genes coding for proteins involved in iron transport

**Table 3:** Iron regulated genes that are common between *A. pleuropneumoniae (App) *and *P. multocida (Pm)*

*App *Gene ID	Gene	*Pm *ORF	Description
*Up-Regulated genes*			
ap1453	*omp64*	576	CopB homolog, heme-hemopexin utilization protein C
ap2032	*lldD*	288	l-lactate dehydrogenase
ap0294	*yfeB*	399	chelated iron transport, ATP binding protein
ap0295-ap0296	*yfeA*	400	chelated iron transport, periplasmic binding protein
ap1739	*exbB*	1186	energy transducing protein
ap0145	*yfeC*	398	chelated iron transport, membrane protein
ap0726	*hlyX*	668	fnr-like transcriptional regulator
ap0144	*yfeD*	129	chelated iron transport, membrane protein
ap1175-ap1176	*hgbA*	741	hemoglobin-bindin protein precursor
ap1740 ap0082	*tonB1 tonB2*	1188	energy transducing protein
ap0755	*aroA*	839	conserved hypothetical protein
ap1738	*exbD*	1187	biopolymer transport protein
ap0286	*nagB*	875	conserved hypothetical protein
ap1505	*HI1275*	656	tellurite resistance protein TehB
ap1363	*fldA*	353	flavodoxin
			
*Down-Regulated genes*			
ap0108	*nrfA*	1792	nitrate reductase cytochrome c552
ap1470 ap1698	*dcuB1 dcuB2*	1434	anaerobic C4-dicarboxylate membrane transporter
ap0169-ap0173	*aopA, nqrBCDE*	1331	NADH: ubiquinone oxydoreductase
ap1937	*fumC*	823	fumarate hydratase class II
ap1588	*napF*	1592	ferredoxin-type protein
ap1822	*atpH*	1491	ATP synthase delta subunit
ap0996-ap0997	*bisC*	408-409	nitrate-inducible formate dehydrogenase-N α subunit
ap0725	*uspA*	1286	universal stress protein A
ap1478	*ptsI*	897	phosphoenolpyruvate PTS system enzyme I
ap1477	*ptsH*	898	phosphocarrier protein Hpr
ap1163	*pflB*	75	formate acetyltransferase
ap0684	*bioD1*	641	probable dethiobiotin synthetase
ap1402	*pgk*	1860	phosphoglycerate kinase
ap1848	*dmsA*	1754	dimethyl sulfoxide reductase
ap0998	*hybA*	407	formate dehydrogenase β subunit
ap0484	*gapA*	924	glyceraldehyde 3-phosphate dehydrogenase
ap1694-ap1692	*frdABC*	201-199	fumarate reductase
ap1132	*adh2*	1453	alcohol dehydrogenase 2
ap1215	*ompW*	331	outer membrane protein W

**Table 4:** Oligonucleotide primers used for microarray results validation with qRT-PCR

#	Gene	Forward Primer	Reverse Primer
	*ackA*	CCTAAAACGGGTGACGAGAA	ACCGATAGCACCGATACTGG
1	*ap1465*	CGTAGCGCGTTCCGAATTAA	AACTGCCGTATTTGTCGTGC
2	*apxIC*	TGGTTATGGGCAAGTTCTCC	CAACTAGCGAGGCAACATCA
3	*ccmC*	ATACGGTTCTATGGCGGTTG	AAACAACACCAAAGCCGAAG
4	*dcuB2*	GGCTTTGAAGGCGTTACACT	GCCGGTAATTGCTCGTCTAA
5	*dmsA*	AACTGTGGTAGCCGTTGTCC	AATGCGGCAAACTGATAACG
	*exbB1*	CCGTTCATTGGGTTATTTGG	ACGGTTAAGGCGAGCAATTA
	*exbD1*	GGGCATTTATTTAGGCGAGA	TGAGTCACAAAGCCTATTTTCG
6	*fetB2*	CCGCTCTTGATATTCCGATG	TTCCAAGCGTTTGTTTGATG
7	*hgbA*	TGAATTTCGGGCAATTATGG	TCCGCTTTCTTCGCACTTAC
8	*NMB1668*	AAACGGATTTCGGCATACAC	CGTACCGGAGAACATTTCGT
9	*nrfA*	AAGAAAAACCGGCTCAAACA	ATAACCCGCCCATAACACAA
10	*nrfC*	GCACCCGTAGAGACTTCGTC	GCCTTCCGGTACTTTGTTTG
11	*nrfE*	CCGTTTGAGCGTAGTTTTCC	ATTGTCCAAGGTCGAATCCA
12	*omp64*	GCGGACAGTAAGCCTGAAAC	TGTTGTCGCATTTGAACCAC
13	*ompW*	GGCGAAGTGGCAAAAGTAAA	CAACACCTAAATTCGCATCG
14	*pepD*	GGCGCAAAAGTAGCATTCTC	TTGTCGGTCCGATAGAAACC
15	*PM0741*	GGCTCGGATTCATTTACCAC	AATAGACCGCATCCAGCTTC
	*tbpA*	ATTGGCAACCATCGGATTTA	GCACCTAAGCGATCACGAGT
16	*tonB1*	CTCCCTTGGTGCTGGTTATG	AATTTTTGCCGGTTGATACG
17	*torA*	GAATTTCCTTGTGCCGAGAG	GCTTCGCCGTATACCAAGTC
